# Supplementary material for: NEIL1 and NEIL2 DNA glycosylases protect neural crest development against mitochondrial oxidative stress
Source: eLife. 2019 Sep 30;8:e49044. doi: 10.7554/eLife.49044 (PMC6768664; doi:10.7554/eLife.49044)
Supplement: Supplementary file 6. [file elife-49044-supp6.docx]

**Supplementary file 6: Oligonucleotides used in this study.**

Morpholino antisense oligonucleotides (MO, Gene Tools)

| **Name** | **Sequence (5’→3‘)** |
| --- | --- |
| Control MO | CCTCTTACCTCAGTTACAATTTATA |
| *neil2* MO | GTCTGACTGTGGGACCTTCCGGCAT |
| *tp53* MO | GCCGGTCTCAGAGGAAGGTTCCATT |
| *apex1* MO | CTTCTTCCCTCTCTTGGGCATTTTC |

Oligonucleotides (Sigma) used to clone gRNA sequences into pX330-U6-Chimeric_BB-CBh-hSpCas9

| **Name** | **Orientation** | **Sequence (5’→3‘)** |
| --- | --- | --- |
| *mmNeil1_P1_1* | Up  Low | CACCGGGAGGAACAGGTTTGTAAG  AAACCTTACAAACCTGTTCCTCCC |
| *mmNeil1_P1_2* | Up  Low | CACCGATGTCAGGATCCTTCCAGC AAACGCTGGAAGGATCCTGACATC |
| *mmNeil2_P1_1* | Up  Low | CACCGCTGTGTGGTTAAGGCTGAG  AAACCTCAGCCTTAACCACACAGC |
| *mmNeil2_P1_2* | Up  Low | CACCGCTGTGAAGAATGTCTGAAA  AAACTTTCAGACATTCTTCACAGC |
| *mmNeil3_V1_1* | Up  Low | CACCGATTTCAGCTCAAAAAGTAT AAACATACTTTTTGAGCTGAAATC |
| *mmNeil3_V1_2* | Up  Low | CACCGCCAGTGCTAGATCCACCGA  AAACTCGGTGGATCTAGCACTGGC |
| *mmApex1_D209_1* | Up  Low | CACCGTGCCCGCTAAAAGTCTCTTA  AAACTAAGAGACTTTTAGCGGGCAC |
| *mmApex1_D209_2* | Up  Low | CACCGAAGATCCGGTCCAAGGCTCT AAACAGAGCCTTGGACCGGATCTTC |
| *mmTdg_N151_1* | Up  Low | CACCGTAAAACTCGCTCTGTAGACC AAACGGTCTACAGAGCGAGTTTTAC |
| *mmTdg_N151_2* | Up  Low | CACCGCAACCTCTGTGCTTGCTCA AAACTGAGCAAGCACAGAGGTTGC |

gRNA sequences are underlined.

CRISPR Genotyping PCR pimers (Sigma)

| **Gene** | **Direction** | **Sequence (5’→3‘)** |
| --- | --- | --- |
| *mmNeil1* | Forward  Reverse | TGTTGGGTGTGTGCGCTATC ACACCCCTCAAGTTCTTGCC |
| *mmNeil2* | Forward  Reverse | TGTCTGGGGCATCCTGATTC  GTGGTGGTGCACGCCTTTAC |
| *mmNeil3* | Forward  Reverse | CAATGATCTTAAGTGACCCC TGGGAGCATATGCTAATTGC |
| *mmApex1* | Forward  Reverse | CAAGATATGCTCCTGGAATG  AAGATGGTTCTAGCCATGCC |
| *mmTdg* | Forward  Reverse | AAGATGTGGACCACCTTGTC AACCAACCAGAACAGGGTG |

Oligonucleotides (IDT) for abasic site quantification

| **Name** | **Sequence (5’→3‘)** |
| --- | --- |
| 40mer | AGAGTCTCCCCGCTAGGATC**C**GGCCCCGCCCAGCGTCTTG |
| 40mer_U | AGAGTCTCCCCGCTAGGATC**U**GGCCCCGCCCAGCGTCTTG |
| 40mer_5hU | AGAGTCTCCCCGCTAGGATC**5hU**GGCCCCGCCCAGCGTCTTG |
| 40mer_complementary | CAAGACGCTGGGCGGGGCCGGATCCTAGCGGGGAGACTCT |

Quantitative RT-PCR primers (Sigma) and UPL probe numbers (Roche)

| **Gene** | **Direction** | **Sequence (5’→3‘)** | **UPL probe** |
| --- | --- | --- | --- |
| *mmTbp* | Forward  Reverse | GGGGAGCTGTGATGTGAAGT  CCAGGAAATAATTCTGGCTCA | 97 |
| *mmOct4* | Forward  Reverse | AATGCCGTGAAGTTGGAGAA  CCTTCTGCAGGGCTTTCAT | 95 |
| *mmNanog* | Forward  Reverse | GCCTCCAGCAGATGCAAG  GGTTTTGAAACCAGGTCTTAACC | 25 |
| *mmKlf4* | Forward  Reverse | CGGGAAGGGAGAAGACACT  GAGTTCCTCACGCCAACG | 62 |
| *mmGata6* | Forward  Reverse | GGTCTCTACAGCAAGATGAATGG TGGCACAGGACAGTCCAAG | 40 |
| *mmEomes* | Forward  Reverse | ACCGGCACCAAACTGAGA  AAGCTCAAGAAAGGAAACATGC | 9 |
| *mmPax6* | Forward  Reverse | GTTCCCTGTCCTGTGGACTC  ACCGCCCTTGGTTAAAGTCT | 78 |
| *mmPax3* | Forward  Reverse | GCCCACGTCTATTCCACAA  GAATAGTGCTTTGGTGTACAGTGC | 69 |
| *mmCcng1* | Forward  Reverse | TTAGTAGGCCTGTCGGATCG AGCAGTTTCTGAGAGTCAGTTGTC | 108 |
| *mmNestin* | Forward  Reverse | TCCCTTAGTCTGGAAGTGGCTA  GGTGTCTGCAAGCGAGAGTT | 67 |
| *mmSox1* | Forward  Reverse | GTGACATCTGCCCCCATC  GAGGCCAGTCTGGTGTCAG | 60 |
| *mmPax2* | Forward  Reverse | AGGCTTGGAGATTCAGCAAC  AACTAGTGGCGGTCATAGGC | 74 |
| *mmHoxa2* | Forward  Reverse | GAAGGCGGCCAAGAAAAC  CATCAGCTATTTCCAGGGATTC | 70 |
| *mmTfapb2* | Forward  Reverse | CAGTGACCTGCACTCCAGAA  GCCAGCAGATCCGTAAATTC | 49 |
| *mmNeurog1* | Forward  Reverse | GACCTGTCCAGCTTCCTCAC  TGGAGGCTAGGGGCTGTAG | 101 |
| *mmMdm2* | Forward  Reverse | TGTTTGGAGTCCCGAGTTTC  AGCCACTAAATTTCTGTAGATCATTG | 99 |
| *mmSesn2* | Forward  Reverse | ACATCCACTGCGTCTTTGG CGTCTTGATATAGATTTTGAGGTTCC | 17 |
| *mmEda2r* | Forward  Reverse | TGTTGTTGCTACACGTGGAAGT TTTTCAAGGCAGTTGTCACG | 109 |
| *mmNeil1 (Exon 2)* | Forward  Reverse | TGTAAGGGGCTGGTATTTGG  TCGGGCTAAAGCTGAGATGT | 108 |
| *mmNeil2 (Exon 2)* | Forward  Reverse | CTGCCGCCTTTCAGTCTCT  TCTGGATCAAACCGAAGGAA | 1 |
| *mmNeil3 (Exon 1)* | Forward  Reverse | CTTGCAACTAGCAGGGGTTC  CCCGGATCTTCTCTCCATTC | 31 |
| *mmActB* | Forward  Reverse | AGGGCTATGCTCTCCCTCA  CACGCTCGGTCAGGATCT | 20 |
| *mm-mt-CytB* | Forward  Reverse | TTATTATCGCGGCCCTAGC  CCTGTTGGGTTGTTTGATCC | 69 |
| *mm-mt-nd5* | Forward  Reverse | AGCATTCGGAAGCATCTTTG  TTGTGAGGACTGGAATGCTG | 31 |
| *mm-mt-nd6* | Forward  Reverse | TGGTTTGGGAGATTGGTTG  CACAACTATATATTGCCGCTACCC | 12 |
| *mm-mt-co1* | Forward  Reverse | CAGACCGCAACCTAAACACA  TTCTGGGTGCCCAAAGAAT | 25 |
| *mmSlc25a42* | Forward  Reverse | CTGCGAGAGGATGCTGAAG  GAGCTGAGCACTTGCCTGT | 20 |
| *hsNEIL1* | Forward  Reverse | GACCAAGCTGCAGAATCCAG  TCTCTGACCCGTAGCCCCTG | 67 |
| *hsNEIL2* | Forward  Reverse | GGGGCAGCAGTAAGAAGCTA GGAATAATTTCTTTCCATGGACCT | 1 |
| *X. laevis histone h4* | Forward  Reverse | GCGGGATAACATTCAGGGTA  GCTTGACTCCCCCTCTCC | 70 |
| *X. laevis tp53* | Forward  Reverse | AGTCACCTGATGCGAGTGG  CCCGCTATTTACATCCTCCA | 80 |
| *X. laevis ccng1* | Forward  Reverse | TTTGCCAGCTGAACTCACTC CAATCAAACGTAAACCACAAGC | 11 |
| *X. laevis eda2r* | Forward  Reverse | CCCATGGTTTGATATGATTGG TCAATCTCAAAATGTAGTGTGTGC | 63 |
| *X. laevis aen* | Forward  Reverse | TTTGCCAGCTGAACTCACTC  CAATCAAACGTAAACCACAAGC | 33 |
| *X. laevis riok3* | Forward  Reverse | AAACATGACGAGGTGGTGTG  CTGGAATTCTGGGGCAAA | 4 |
| *X. laevis mt-nd1* | Forward  Reverse | CACGATTTCGATATGATCAACTTATAC  ATGTCATAATGTTATGGCTAGTGTGAT | 69 |
| *X. laevis mt-nd4* | Forward  Reverse | CCAAATCTCTACCCCTTTGCTA  CGTGAGATTGGTTCGTTTGA | 7 |
| *X. laevis mt-nd5* | Forward  Reverse | TGCCTCAAATATGCTTCCAA  GATTAATCCTGTAACCGAAACGA | 27 |
| *X. laevis mt-Co3* | Forward  Reverse | CCCTACATATGAATTAGGGGAATG  TCAAATGGGTTTAATGGGGTA | 49 |

mm, murine; hs, human

Quantitative-PCR primers (Sigma) for detection of mtDNA damage

| **mt-fragment** | **Direction** | **Sequence (5’→3‘)** | **Length (bp)** |
| --- | --- | --- | --- |
| 1 | Forward  Reverse (short)  Reverse (long) | TAAATTTCGTGCCAGCCACC  GTTGACACGTTTTACGCCGA  ATGCTACCTTTGCACGGTCA | 72  1739 |
| 2 | Forward  Reverse (short)  Reverse (long) | GGCGGTAGAAGTCTTAGTAGAGAT  TGGCTGAGTAAGCATTAGACTGT  CTAGGGAGGGGACTGCTCAT | 136  2334 |
| 3 | Forward  Reverse (short)  Reverse (long) | AAGAAGGAGCTACTCCCCACC  AGCTTATATGCTTGGGGAAAATAGT  GTTGACACGTTTTACGCCGA | 139  1308 |
